# Late-Onset Autoimmune Lymphoproliferative Syndrome in a Costa Rican Woman

**DOI:** 10.7759/cureus.50226

**Published:** 2023-12-09

**Authors:** Alberto Alfaro-Murillo, Oscar Correa-Jimenez, Jorge González-Chapa, Tibisay Viloria-González, Melvin Calvo-Solís, Silvia Maradei-Anaya

**Affiliations:** 1 Internal Medicine and Clinical Immunology, Hospital San Juan de Dios, San José, CRI; 2 Allergy and Immunology, Hospital Fundación Neumológica (Pulmonological Foundation Hospital), Bogotá, COL; 3 Rheumatology, University of Washington, Seattle, USA; 4 Pathology, Hospital Calderón Guardia, San José, CRI; 5 Pediatric Immunology and Rheumatology, Hospital Nacional de Niños (National Children's Hospital), San José, CRI; 6 Human and Clinical Genetics, Biotecgen SAS, Bogotá, COL

**Keywords:** length of spleen, autoimmune hemolytic anemia (aiha), auto immune, lymph node-negative, atypical lym

## Abstract

Autoimmune lymphoproliferative syndrome (ALPS) is a primary immune regulatory disorder (PIRD). This disease usually develops during childhood. However, atypically, some cases may have their onset in adulthood. We report the case of a 44-year-old woman with a history of autoimmune hemolytic anemia at 33 years old. The patient presented due to asthenia and a large, painful lymph node in the left axillary region for the last four months. Enlargement of the axillary and inguinal lymph nodes was found by mammography, breast, and abdominal ultrasounds.

An excisional biopsy of the axillary lymph node conglomerate did not document immunophenotypical alterations of T or B lymphocytes but showed progressive transformation of germinal centers with reactive follicular hyperplasia. The lymph node cytometry did not show a malignant phenotype. The immunological work-up documented IgG and IgA hypergammaglobulinemia and slightly decreased IgM; the B cell immunophenotype documented a slight increase in CD21low B cells and decreased memory B cells. The blood count was normal. The T cell compartment evidenced 27% CD3^+^/αβ^+^/γδ^-^/CD4^-^/CD8^-^ of the total T CD3^+ ^cells and 15% of the total lymphocytes. A pathogenic heterozygous variant in the *FAS* gene, exon 9, c.785T>A (p.Ile262Asn), was documented. This variant has not been previously described.

This case highlights the importance of considering the diagnosis of ALPS even in adulthood. Genetic conditions such as incomplete penetrance or variable expressivity that depend on factors that are not entirely clear in ALPS, such as epigenetics and environmental factors, among others, could generate the onset of this disease in adulthood in a smaller number of patients.

## Introduction

Autoimmune lymphoproliferative syndrome (ALPS) is a primary immune regulatory disorder (PIRD) classically characterized by autoimmunity and malignant or benign lymphoproliferation [1]. The first gene associated with this disorder was TNFRSF6 (FAS) [2]. Since then, different variants of this gene have been described as responsible for about 72% of ALPS cases [3]. The *FAS *gene encodes a cell surface receptor that, upon stimulation, induces programmed cell death [4]. The patients usually have a high number of double-negative T cells (CD3+CD4-CD8- T cell receptor αβ+), polyclonal elevation of gammaglobulin, and an increase in IL-10 [5]. In about 80% to 99% of patients, there are chronic noninfectious lymphadenopathies, splenomegaly, and autoimmune cytopenias. This disease typically develops during childhood, although there are few reported adult-onset cases [6].

## Case presentation

A 44-year-old woman consulted our internal medicine department due to asthenia which began four months before presentation. She also referred to a large, painful lymph node in the left axillary region. The only notable finding in her past medical history was a refractory IgG warm autoimmune hemolytic anemia; consequently, she required a splenectomy 10 years ago. Subsequently, she did not present any other clinical relapse of that disease. Regarding her family history, she indicated that her youngest son, who is 8 years old, is in clinical studies due to lymph node enlargement and Evans syndrome.

**Figure 1:**
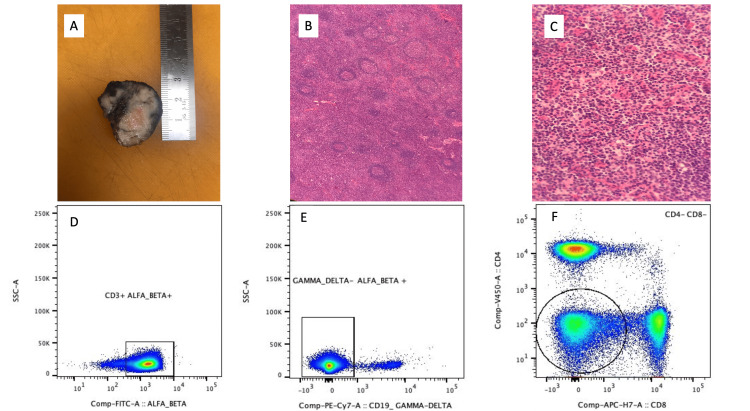
Lymph node histology and peripheral blood flow cytometry of double negative T cells A: Macroscopic enlargement of axillary lymph node; B-C: Histological findings of progressive transformation of germinal centers with reactive follicular hyperplasia; D-F: CD3^+^/αβ^+^/γδ^-^/CD4^-^/CD8^- ^peripheral blood flow cytometry

A mammogram and breast and abdominal ultrasounds revealed enlargement of the axillary and inguinal lymph nodes. Radiological studies reported a breast imaging reporting and data system (BI-RADS)-5 classification, compatible with a high suspicion of breast cancer. Due to this radiological finding and the possibility of metastatic lymph node enlargement, an excisional biopsy of the axillary lymph node conglomerate was performed (Figure 1), which ruled out metastatic disease. Special stains for fungi and tuberculosis were negative. A slight increase in CD8+ T cells, progressive transformation of germinal centers with reactive follicular hyperplasia, and a slight increase in lymphoproliferative index with a moderate number of lymphocytes with Epstein Barr (EBER+) were observed. Non-Hodgkin lymphoma was ruled out on lymph node biopsy by performing a lymphoma screening tube flow cytometry with the following monoclonal antibodies: CD19/CD45/CD3/CD8/CD4/LAMBDA/KAPPA.

Initial laboratory work-up was resumed (Table 1). Negative antinuclear antibodies (ANA), negative antineutrophil cytoplasmic antibodies (ANCA)-C and ANCA-P, normal serum complement C3 and C4, IgG and IgA hypergammaglobulinemia, and a slightly decreased IgM were documented. Positive IgG anti-Epstein-Barr virus (EBV) and negative IgM anti-EBV were documented. The EBV DNA in the plasma was not detected. Both HIV and pneumococcal infections were ruled out in our patient. She received the pneumococcal conjugate vaccine (PCV)-13 and pneumococcal polysaccharide vaccine (PPSV)-23 vaccines. The T cell immunophenotype distribution in peripheral blood was normal, while the B cell immunophenotype documented only a slight increase in CD21low B cells and decreased memory B cells (Table 2). 

**Table 1:** Immunological tests The altered values are highlighted in bold. ANA: Antinuclear antibodies, IFA: Indirect immunofluorescence assay, ANCA: Anti-neutrophil cytoplasmic antibodies, EBV: Epstein Barr virus, EA: Early antigen, VCA: Viral capsid antigen, EBNA: Epstein Barr nuclear antigen

Laboratory test	Patient value	Reference value for age (units)
ANA	Negative	>1:80 IFA
ANCA C, ANCA P	Negative	Negative (IU/mL)
Complement C3	119	90-180 (mg/dL)
Complement C4	21	10-40 (mg/dL)
IgG	2005	700-1600 (mg/dL)
IgG1	1016	382-928 (mg/dL)
IgG2	245	241-700 (mg/dL)
IgG3	22	21.8-176 (mg/dL)
IgG4	77	3.9-86.4 (mg/dL)
IgA	452	70-400 (mg/dL)
IgM	30	40-230 (mg/dL)
Vitamin B12	1000	200-650 (pg/mL)
HIV	0.10	<1.00 (S/CO)
EBV EA IgG	>150	Positive >40 (U/mL)
EBV VCA IgG	>750	Positive >20 (U/mL)
EBV EBNA IgG	<3	Positive >20 (U/mL)
EBV VCA IgM	<10	Positive >40 (U/mL)
EBV-DNA	<65	<65-526.314 (copies/mL)

**Table 2:** T and B cell immunophenotype The altered values are highlighted in bold. TEMRA: Terminally differentiated effector memory

T cells	Patient values		Reference values for age (cells/uL)
	%	Absolute (cells/uL)	
Normal number of CD3^+^CD4	26	600	400-1330
CD4^+ ^naive (CD27^+^CD45RA^+^)	53	318	87-796
CD4^+ ^total memory (CD45RO^+^)	39	234	231-600
CD4^+ ^effector T cells	61	366	66-900
TEMRA CD4^+^	7	42	1-130
Normal number of CD3^+^CD8^+^	19	438	185-1024
CD8^+ ^naive (CD27^+^CD45RA^+^)	42	184	37-484
CD8^+ ^total memory (CD45RO^+^)	35	153	20-309
CD8^+ ^effector T cells	64	280	45-564
TEMRA CD8^+^	22	96	17-274
B cells			
Total CD19^+^	26	600	80-399
Transitional B cells CD20+CD10+CD27^-^	23	48	1-64
B naive cells CD20^+^CD10^-^CD27^-^IgM^+^IgD^++^	60	124	26-244
Total memory B cells CD19^+^CD20^+^CD27^+^	8.5	18	18-120
Memory switched B cells CD20^+^CD27^+^IgM^-^IgD^-^	1	2	6-49
CD21low B cells	6.4	13	0-9
Plasma cells CD19^+^CD38^++^ CD27^++^	1.4	3	0-3

After the clinical analysis and differential diagnosis, the clinical suspicion of ALPS was raised. A double-negative T cell flow cytometry documented 27% CD3+/αβ+/CD4-/CD8- of the total T CD3+ cells and 15% of the total lymphocytes. Clinical exome sequencing (SOPHiA DDM™ Clinical Exome Solution, SOPHiA Genetics, Lausanne, Switzerland) revealed a heterozygous variant in the *FAS* gene, exon 9, c.785T>A (p.Ile262Asn). This finding was confirmed with Sanger sequencing. The same variant was found in the patient's son. In Costa Rica, there is no possibility of performing functional tests on the *FAS* gene.

## Discussion

This case highlights the importance of considering the diagnostic possibility of ALPS in adult patients with chronic lymphoproliferation and cytopenias, even in cases like this where malignant etiology is considered. Classically, the onset of this inborn error of immunity is in childhood. However, genetic conditions such as incomplete penetrance or variable expressivity that depend on factors that are not entirely clear in ALPS, such as epigenetics and environmental factors, among others, could generate the onset of this disease in adulthood in a smaller number of patients [6]. The immune dysregulation caused by ALPS is characterized by the presence of chronic lymphadenopathy, splenomegaly, and autoimmune cytopenias [7]. These findings could help guide the clinician in making an ALPS diagnosis.

It is important to emphasize that Evans syndrome could be present in about 50% of ALPS patients [8]. After lymphadenopathy, autoimmunity is the second most common manifestation in ALPS patients; therefore, they might be erroneously diagnosed with systemic lupus erythematosus (SLE) or mixed connective tissue disease [9]. Similarities between ALPS and SLE, such as hypergammaglobulinemia/autoantibodies, mediated FAS apoptosis defects, and elevated double-negative T cells, might hinder the diagnosis in these patients [9].

The fact that our patient was splenectomized is important due to the possibility that the ALPS onset was probably developed at that time, especially since it has been described that more than 80% of ALPS patients develop autoantibodies to red blood cells [10]. Furthermore, in ALPS-splenectomized patients, the increased risk of pneumococcal sepsis has been described at approximately 29% when compared to the incidence of sepsis among post-splenectomy patients without ALPS, which is approximately 3% [11]. Although the rate of infections due to encapsulated bacteria is higher during the first three years after splenectomy, the risk can remain for decades, especially for Streptococcus pneumoniae infections.

The double-negative T cell population is one of the main ALPS parameters to evaluate to establish the diagnosis. The CD3+/TCRαβ+/CD4-/CD8- cells in healthy individuals represent about 1% of all T cells. It has been considered that an elevation of these double-negative T cells >1.5% of total lymphocytes or >2.5% of CD3+ lymphocytes represents pathogenic levels that require extensive evaluation [12]. In addition, during the extensive immunological work-up study, the typical findings were a slight increase in CD21low B cells and decreased memory B cells. Both findings have been described in chronic inflammation and exhaustion of B cells secondary to primary immune regulatory disorders, with descriptions in different conditions such as CTLA4 haploinsufficiency, LRBA deficiency, activated PI3K delta syndrome, and common variable immunodeficiency, among others [13].

Although the first-line treatment for ALPS is steroid therapy with some other immunosuppressive drug, in this case, we decided to choose expectant management because the patient was asymptomatic. Also, given her medical history, anti-pneumococcal antibody titers were monitored to determine the need for revaccination.

The c.785T>A (p.Ile262Asn) variant in the *FAS* gene has 1 entry in ClinVar, where it is listed per the American College of Medical Genetics and Genomics (ACMG) guidelines as likely pathogenic. The non-conservative amino acid substitution occurs at a position within the critical death domain, which is likely to impact secondary protein structure as the Ile and Asn residues differ in charge, polarity, and other physicochemical properties. Also, in silico analysis in many different bioinformatic tools (BayesDel addAF, BayesDel noAF, MetaLR, MetaRNN, MetaSVN, REVEL, etc.) predicts this variant is most likely damaging to the protein structure and/or function (calibrated predictions: pathogenic 5/20, VUS 12/20, benign 3/20; indicative predictions: damaging/pathogenic 10/14, medium/neutral 3/14, tolerated 1/14). This variant does not have a gnomAD genome entry, meaning no gnomAD frequency for either healthy or disease-related individuals, but its locus is covered in gnomAD genomes at 20X in at least 97.59% of samples registered. To consider, there is evidence supporting missense variants in nearby residues that are related to FAS-associated disorders [14].

Due to the above-mentioned factors, we believe this variant to be pathogenic. However, there is not yet sufficient evidence according to the current ACMG guidelines. Functional studies with the current patient would definitely be useful. Since there is neither accurate nor sufficient information in the literature on this variant, the clinical phenotype that it generates is unknown so far. The early onset in the patient's son in contrast to the late onset in our patient is a reflection of the variable expressivity, which is yet to be well elucidated specifically in this variant.

## Conclusions

We report a likely pathogenic variant in the *TNFRSF6 *gene associated with a clear clinical phenotype of ALPS that was identified during an extensive diagnostic workup study of suspected malignant lymphoproliferation in an adult patient, in whom previous medical history, as well as family history, shed light on an inborn error of immunity as a possible etiology. It is imperative to keep in mind that although ALPS is an immunohematologic disease with classic onset in pediatrics, there is a possibility of onset in adulthood. The coexistence of autoimmune cytopenias, splenomegaly, chronic lymphoproliferation, and even the development of lymphoma in the presence of increased double-negative T cells may be red flags for identifying a patient with ALPS.
